# Technology Implementation for Mental Health End Users: A Model to Guide Digital Transformation for Inpatient Mental Health Professionals

**DOI:** 10.2196/40429

**Published:** 2023-04-06

**Authors:** Jessa Lin Westheimer, Nidal Moukaddam, Jan A Lindsay, Ashutosh Sabharwal, Bijan Najafi, Peter A Iacobelli, Robert J Boland, Michelle A Patriquin

**Affiliations:** 1 Research Department The Menninger Clinic Houston, TX United States; 2 Department of Psychiatry and Behavioral Sciences Baylor College of Medicine Houston, TX United States; 3 Adult Outpatient Services Ben Taub Hospital Houston, TX United States; 4 Michael E DeBakey VA Medical Center Houston, TX United States; 5 Department of Electrical and Computer Engineering Rice University Houston, TX United States; 6 Michael E DeBakey Department of Surgery Baylor College of Medicine Houston, TX United States; 7 The Menninger Clinic Houston, TX United States

**Keywords:** digital transformation, user-centered design, innovation, implementation science, user acceptability, wearables, mental health, implementation, technology implementation

## Abstract

Digital transformation is the adoption of digital technologies by an entity in an effort to increase operational efficiency. In mental health care, digital transformation entails technology implementation to improve the quality of care and mental health outcomes. Most psychiatric hospitals rely heavily on “high-touch” interventions or those that require in-person, face-to-face interaction with the patient. Those that are exploring digital mental health care interventions, particularly for outpatient care, often copiously commit to the “high-tech” model, losing the crucial human element. The process of digital transformation, especially within acute psychiatric treatment settings, is in its infancy. Existing implementation models outline the development of patient-facing treatment interventions within the primary care system; however, to our knowledge, there is no proposed or established model for implementing a new provider-facing ministration tool within an acute inpatient psychiatric setting. Solving the complex challenges within mental health care demands that new mental health technology is developed in concert with a use protocol by and for the inpatient mental health professional (IMHP; the end user), allowing the “high-touch” to inform the “high-tech” and vice versa. Therefore, in this viewpoint article, we propose the Technology Implementation for Mental-Health End-Users framework, which outlines the process for developing a prototype of an IMHP-facing digital intervention tool in parallel with a protocol for the IMHP end user to deliver the intervention. By balancing the design of the digital mental health care intervention tool with IMHP end user resource development, we can significantly improve mental health outcomes and pioneer digital transformation nationwide.

## Background

Digital transformation in mental health care has reshaped care delivery over the last few years and has been catalyzed particularly due to the global COVID-19 pandemic. Digital transformation is the adoption of digital technologies by an entity in an effort to increase operational efficiency [1]. In mental health care, digital transformation entails technology implementation to improve the quality of care and clinical outcomes. Digital transformation makes possible an expanded “full continuum of care” for inpatient psychiatry, which is desperately needed.

Earlier studies have shown that the success of a digital transformation initiative is predicated on transparency, user-centered design, and iteration [2]. Additionally, it is important to balance the design of the digital health care intervention with end user resource development [1,3,4]. Other implementation models for digital transformation have been proposed [4-8]. For example, the Accelerated Creation-to-Sustainment (ACTS) model outlines a framework for implementing a sustainable digital mental health intervention in a real-world treatment setting that emphasizes rapid, iterative, user-centered design [5]. This model describes an end-to-end process for accelerating the research-to-practice pipeline for digital mental health care innovations. While the ACTS model importantly incorporates user-centered design and sustainable implementation, this model is intended to develop a patient-facing treatment intervention within the primary care system. To our knowledge, there is not yet a comparable model for implementing a new provider-facing ministration tool within an acute inpatient psychiatric setting.

Inpatient mental health professionals (IMHPs) are vulnerable to experiencing burnout [9,10]. Sources of stress and burnout unique to the roles of IMHPs include demanding therapeutic relationships, difficult and sometimes violent patients, and patient safety management (where the risk for suicide and self-harm is constant) [9,10]. Because of the inpatient setting’s unpredictability, the adaptability of an experienced IMHP is relied on heavily to ensure quality patient care. Digital transformation offers the opportunity to offset the stress and workload of IMHPs by leveraging the continuous availability and predictive power of technology. Importantly, the unique demands for an IMHP demand a unique approach to digitally transforming clinician-facing tools within an inpatient psychiatric setting to ensure their successful implementation and that the tools reduce, not increase, stress and burnout.

**Figure 1:**
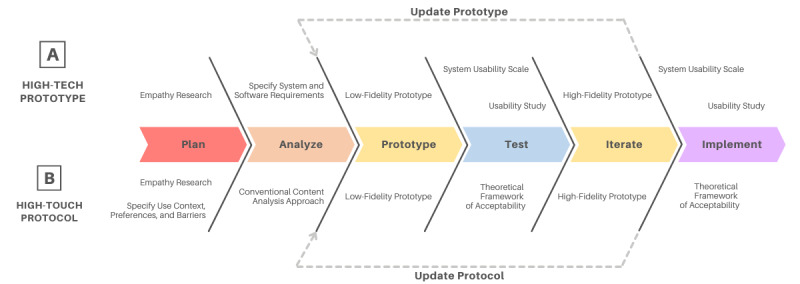
The Technology Implementation for Mental-Health End-Users framework for inpatient mental health care. (A) "High-tech prototype" refers to a technology-based, inpatient mental health professional (IMHP)–facing mental health care innovation or intervention. The top steps outline the development of a high-tech prototype. (B) "High-touch protocol" refers to the human side of mental health care (how the prototype will be used by the IMHPs). The bottom steps outline the development of a high-touch protocol.

Therefore, we propose the Technology Implementation for Mental-Health End-Users (TIME) framework in inpatient mental health care (Figure 1), which outlines the process of developing a prototype of an IMHP-facing intervention (Figure 1A) in parallel with a protocol for an IMHP to deliver the intervention (Figure 1B). Similar to the ACTS model, the TIME framework is grounded in well-defined frameworks for user-centered design, software development, and implementation science. The TIME model also emphasizes participatory design—the active involvement of the end user in the design process—so the ultimate deliverable has inherent buy-in and utility [11,12].

**Figure 2:**
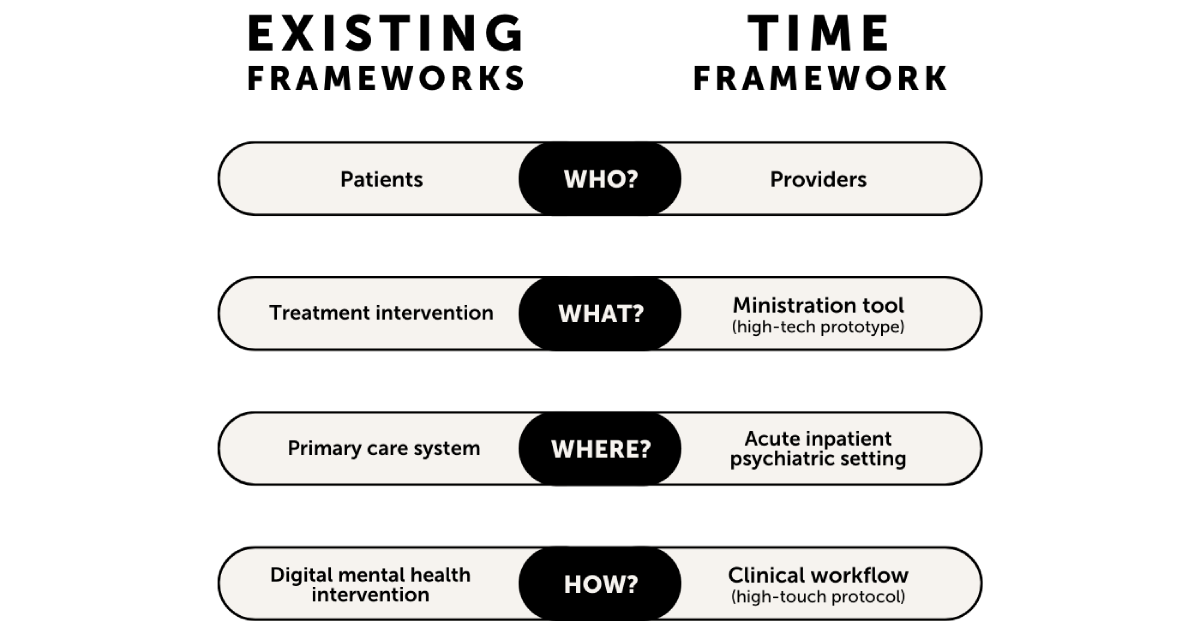
Comparing the Technology Implementation for Mental-Health End-Users (TIME) framework with existing frameworks, such as the Accelerated Creation-to-Sustainment (ACTS) model.

An important feature of the TIME framework is its balance of the “high-tech” prototype with the “high-touch” protocol. Most inpatient psychiatric hospitals rely heavily on “high-touch” interventions, that is, interventions that require in-person, face-to-face interaction with the patient [13]. Conversely, those that are developing digital mental health care interventions often fully commit to the “high-tech” model, losing the crucial human element. By leveraging the continuous availability and predictive power of technology in concert with the adaptability and experience of clinical care providers, we can significantly improve mental health outcomes and pioneer digital transformation. While the term “blended care” refers to the blending of technology and face-to-face treatment for a patient-facing intervention [14], the TIME framework produces a form of “blended ministration” where an IMHP-facing technology is blended with an IMHP-facing implementation blueprint (Figure 2).

## Operational Definitions

Operational definitions are provided in Table 1.

**Table 1:** Operational definitions.

Term	Definition
High-tech	Referring to technological innovations and interventions
High-touch	Referring to the human side of mental health care (clinical care staff interacting directly or face-to-face with the patient)
Wearable [15,16]	Electronic devices that can be worn on or close to the user’s body to monitor vital signs
End user	The final user of the intervention and protocol (in the TIME^a^ model, typically nursing or clinical care staff)
Empathy research [11,17]	The design process of empathizing with the end user of the project through interviews and contextual inquiry

^a^TIME: Technology Implementation for Mental-Health End-Users.

## Challenges

### Technology Acceptance

In the Technology Acceptance Model, an individual’s acceptance of a new technology is determined by its perceived usefulness and perceived ease of use [18]. Therefore, perceived usefulness and perceived ease of use govern individuals’ attitudes toward the new technology and, in turn, their use of it [18]. Therefore, the successful implementation of an IMHP-facing technology in inpatient psychiatric care must consider the perceived use of the IMHP (“will they trust this innovation?”), the perceived ease of use of the IMHP (“is this innovation technologically unambiguous?”), and the attitude of the IMHP (“will this innovation positively contribute to their treatment of patients?”).

Technology acceptance was tested significantly during the global COVID-19 pandemic [19]. Quarantine meant an immediate and massive digital migration, where digital immigrants found themselves thrust into unavoidable technology and unprecedented connectivity [20,21]. By the same token, the demographic landscape of the mental health care system comprises predominantly digital immigrants—generally considered those who are older than 40 years and were born before the “proliferation of digital technologies.” In 2022, the average age of a mental health care professional in the United States is 44 years, with 60% of the professionals being older than 40 years [22]. Thus, designers of digital interventions for mental health must carefully consider usability, inclusivity, and accessibility [23,24].

### Perceived Dispensability of User-Centered Design

User-centered design is an approach to development wherein the intended users of the system (end users) are actively and intentionally involved in its development. There are three major tenets of this framework, defined by Gould and Lewis [25] as follows:

1. Front-load with empathy: understanding the end users’ needs and expectations from the project’s inception ensures *customized solutions*.
2. Measure usability empirically: quantifying the end users’ satisfaction throughout project development ensures *usability*.
3. Design and test iteratively: designing and testing the system iteratively with the end users’ input ensures *acceptability*.

Despite its proven effectiveness in developing digital systems, there is still a widely held belief that applying user-centered design is superfluous to the development life cycle [26]. It is common for developer-driven needs to take priority over those of the user [26,27]. Additionally, there is a stigma that user-centered design is time-consuming and costly to the developer; on the contrary, by involving the intended user throughout the design process, usability problems are identified and resolved before the system is ever deployed, saving time and money [26-28]. By emphasizing empathy research, empirical measures of usability and iterative design based on user-centered design principles increase the likelihood for successful implementation and acceptability. Digital transformation in inpatient psychiatric care will require a shift to the user-driven development of novel technologies and interventions.

### Health Care Disparities and Technology Implementation

Internet and smartphone availability are ubiquitous in the general population. However, this ubiquity is curtailed by the following two primary factors: socioeconomic disparities and mental illness symptoms. A closer look reveals that the first gap is in smartphone ownership and internet access. Of patients discharged from inpatient psychiatric units in the United States, 82% are estimated to have smartphones and internet access [29]; however, in high-income households, that number is close to 100% [30]. This digital divide correlates with socioeconomic and racial disparities. For example, low-income Hispanic and Black youth are less likely to have home or smartphone internet access [31]. So, while digital technologies promise to bring individualized high-tech monitoring, their effect could be limited by a return to underresourced environments upon discharge from an inpatient setting.

The second gap relates to the severity of mental illness symptoms that affect trust in and use of technology. In a survey of individuals with severe mental illness (SMI) on internet and smartphone use during the global COVID-19 pandemic, 61.6% of them were limited users or nonusers of the internet, mostly using phones to communicate with their families and friends [32]. A quarter of the surveyed sample—markedly among older individuals with schizophrenia—reported being weary of internet use for “security concerns.” Thus, while most mobile health interventions report favorable feasibility outcomes, they typically do not have the same success rate in including patients with SMI. Studies that document significant web-based participation among individuals with SMI demonstrate a correlation with positive recovery and positive emotions toward technology [33]. These findings suggest that attitudes of people with SMI meaningfully affect successful digital transformation. There are gaps in our understanding of how delusions, paranoia, and other severe symptoms may impact the scalability of the TIME framework as well, and these are intriguing areas for continued research and development.

## The TIME Framework

### Overview

When we have more technology available than ever before, the entire onus of patient safety should not fall on the IMHP. An important feature of the TIME framework is its balance of the “high-tech” prototype with the “high-touch” protocol. Most psychiatric hospitals rely heavily on “high-touch” interventions, that is, interventions that require in-person, face-to-face interaction with the patient [13]. Those that are exploring digital mental health care interventions, particularly for outpatient care, often copiously commit to the “high-tech” model, losing the crucial human element [34-37]. Solving the complex challenges faced by mental health care providers demands that new mental health technology tools are developed in concert with a use protocol by and for the end user (mental health care provider), allowing the “high-touch” to inform the “high-tech” and vice versa.

**Figure 3:**
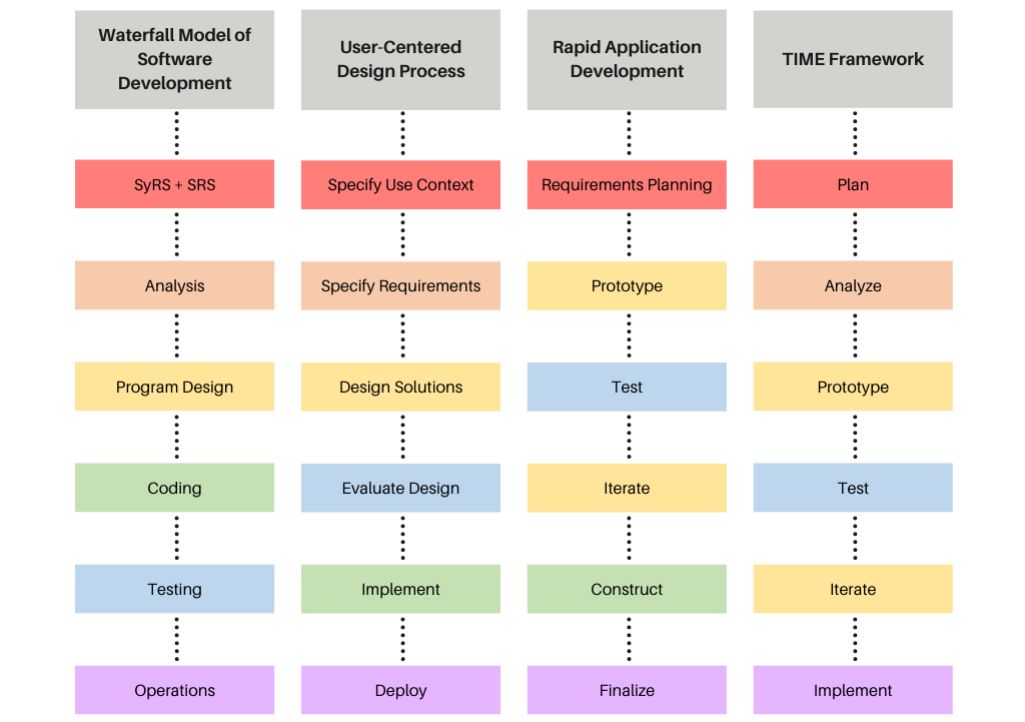
The Technology Implementation for Mental-Health End-Users (TIME) framework is grounded in design processes across disciplines. The TIME framework combines elements from the Waterfall Model of Software Development [38], the user-centered design process [11,17], and rapid application development [39]. Many of these processes contain corresponding steps and are color coded as follows: planning steps describing information gathering for the project, including empathy and user-based research (red); analysis steps describing how any information gathered in a planning step is further evaluated to define the project’s scope and requirements (orange); prototype or iterate steps describing the iterative process of prototyping the design solution (yellow); construct steps describing the creation of a final deliverable (green); test steps describing the evaluation of the design solution and collection of feedback from end users (blue); and implementation of steps describing how the design solution is implemented (purple).

High-tech interventions and their corresponding high-touch protocols must be developed alongside each other to ensure usability and end user acceptability. Thus, we have defined a model—the TIME framework—for developing novel IMHP-facing tools and their clinical workflows alongside each other (Figure 1). This model is grounded in established frameworks for user-centered design and digital technology development (Figure 3).

### Phases of the TIME Framework

#### Plan Phase

At the core of the *Plan* phase of the TIME framework is empathy research [11,17]. During this phase, interviews are conducted with the IMHP end user of the mental health care tool. Prior to this phase, the clinical providers’ problem has been identified. The goal of these interviews will be to understand the needs and expectations of the IMHP end user for a high-tech solution to the clinical problem. Importantly, this entails building an in-depth understanding of the current clinical workflows and characterizing the distinct needs of different types of IMHPs. For instance, a day shift mental health nurse would have different responsibilities from those of a night shift mental health nurse (eg, nighttime safety checks). Likewise, barriers and preferences for the use protocol will be defined, so careful consideration must be made for institutional policies and regulation (ie, crisis intervention protocols, such as seclusion and restraint, hospital documentation guidelines, and others). Empathizing at the onset operationalizes the end users’ needs in the solutions we design.

#### Analyze Phase

The feedback from empathy research will be analyzed to specify prototype requirements. Understanding the functional and emotional needs of the end user is essential to human-centered design. In the *Analyze* phase*,* qualitative discourse is anatomized to scope both the technical requirements for the IMHP-facing digital intervention and the key features of its use protocol within the context of the inpatient psychiatric hospital setting. In applying the TIME framework, it is possible to have different IMHP end user groups. For example, in designing a new electronic health record system, the end users could be mental health nurses, social workers, psychologists, psychiatrists, researchers, physicians, administrative assistants, and others. Developing a firm understanding of both the unique and overlapping needs of each group during this phase will ensure a more functional prototype. Further, the needs of these mental health professionals differ significantly from those of general medical professionals [40,41]. A common misstep in establishing digital transformation in an inpatient psychiatric setting is the presumption that an intervention for physical health can be superficially adapted to mental health. For instance, among the chief concerns during the implementation of an electronic health record system within a mental health hospital in England was that the technology was “not fit for [a mental health] purpose” and much customization would be required for the preexisting system to meet the needs of their setting [42].

#### Prototype Phase

Before resources are dedicated to developing sophisticated technologies, low-fidelity prototypes of initial designs will be created and evaluated in the *Prototype* phase of the TIME framework. The digital intervention prototype will capture the basic architecture and user flow of the technology. A major benefit of digital transformation is the potential to manage exponentially larger data sets. In an inpatient psychiatric setting, consideration of the end users’ ultimate data needs will inform prototype configuration. Innovations developed in an inpatient psychiatric setting may serve discrete goals for different IMHP end users. For instance, in developing a new data management system for clinical outcomes, a clinician would need item-level responses to outcome measures, while a researcher may only need unit-wide average scores. The use protocol prototype will outline the procedure for using the technology within the context of the IMHP’s role. Similar to the implementation blueprint in the ACTS model, this will be a critical part of facilitating the transition to a usable intervention [5].

#### Test Phase

During the *Test* phase*,* the low-fidelity prototypes will be evaluated by the end users through usability studies adapted for ascertaining qualitative and quantitative feedback to update the design. Key performance indicators (time on task, use of navigation vs search, conversion rate, error rate, and drop-off rate) and adapted system usability scales will be defined to guide quantitative evaluation. To evaluate the prototype’s usability and the protocol’s effectiveness, usability studies will be designed and carried out with simulated practical scenarios in the target inpatient setting. Additional moderated usability studies will be conducted to assess more qualitative feedback about the prototype and the clarity of its use protocol. Testing and feedback review with the IMHP end users are necessary for participatory design and ensuring the usability of the final solution.

#### Iterate Phase

Human-centered design is inherently iterative. Adapting each design iteration to tailor to the preferences and behaviors of the IMHP end user will produce better, more acceptable digital mental health care interventions. During design, developers maintain a high level of control over a prototype; therefore, the evaluation of the prototype’s behavior in a real-world clinical care setting is necessary to make it truly functional in its final state. In the *Iterate* phase, the IMHP end user feedback gathered during the usability studies informs the development of the high-fidelity prototypes.

#### Implement Phase

Applying the iterative process of prototyping and testing, the high-fidelity prototypes are evaluated by way of a usability study again in the *Implement* phase. Repeated evaluations ensure that any changes made are improvements to the overall design and acceptability by the IMHP end user. Additionally, the ultimate goal is to deploy the prototype and protocol in a real-world inpatient treatment setting, which is inherently unpredictable and chaotic. Reevaluating updated designs is helpful for verifying that the IMHPs’ feedback is being captured and the technology can stand up to a variable treatment environment.

The *Test*→*Iterate*→*Implement* sequence is repeated as many times as necessary to achieve an effective solution (digital intervention and its use protocol).

### Hypothetical Example

To clarify the phases of our TIME model, we apply it as a framework for developing a nurse station monitoring dashboard to visualize wearable-based data in real time.

**Figure 4:**
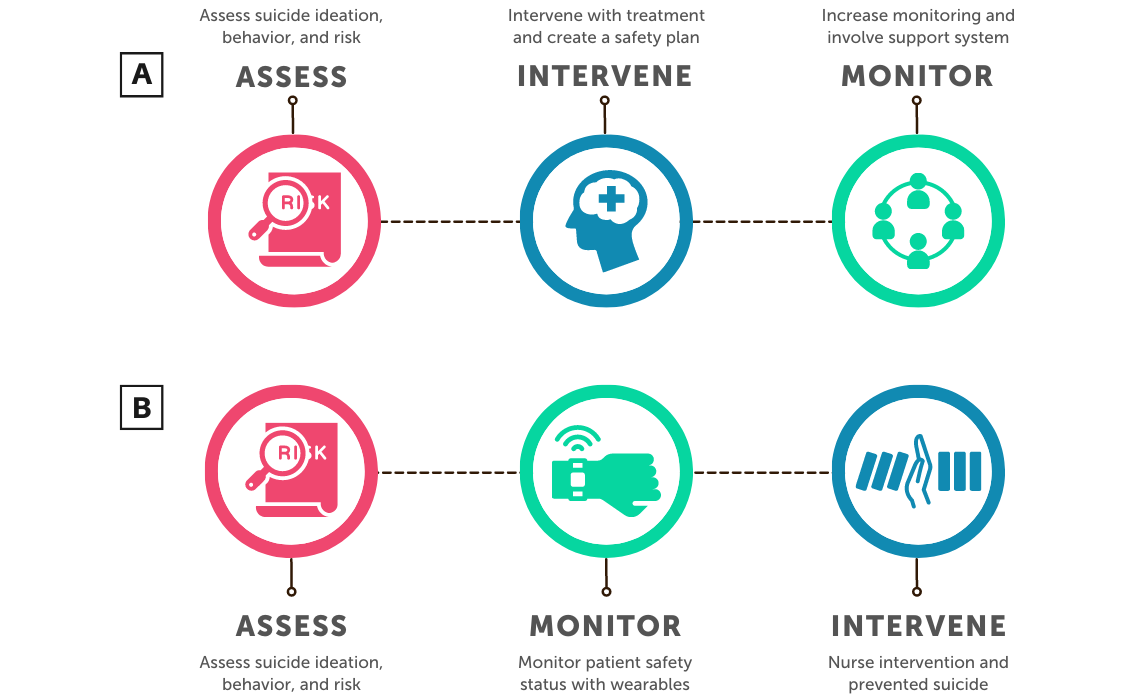
Combining high-tech and high-touch solutions to rearrange the Assess, Intervene, and Monitor for Suicide Prevention (AIM-SP) model. (A) The AIM-SP model [44]. (B) The digitally transformed AIM-SP model incorporating digital technology through remote monitoring can improve suicide prevention outcomes.

In an inpatient hospital setting, there is an average of 56.7 patient suicides a year [43]. Further, 74% of those occur while the patient is undergoing psychiatric treatment. The issue of suicide is pressing. The Assess, Intervene, and Monitor for Suicide Prevention model proposes a clinical management framework to *assess* suicide risk, *intervene* with coping strategies, and *monitor* high-risk patients [44] (Figure 4A). This is a high-touch solution. We propose that by leveraging the continuous availability and predictive power of technology through digital transformation, we may be able to rearrange the Assess, Intervene, and Monitor model as follows: (1) assess suicide risk, (2) monitor patient safety status by using wearable devices, and (3) intervene and prevent suicide every time (Figure 4B). This is a high-tech and high-touch solution. The capitalization on the continuous, noninvasive availability of technology has been described as “digital phenotyping” [13]. We can draw upon wearable technology to provide insight and predictive power for a patient’s status without having to be present in front of them. Not only will this monitoring dashboard display a patient’s current state (awake, asleep, walking, sitting, etc) as measured by the wearable device, but also it will serve as a “digital smoke alarm” for a critical change in the patient’s status [13]. Feedback provided by IMHP end users will inform both the development of the dashboard’s user interface and the crisis intervention protocol when a dashboard alert is issued.

Below, we outline how the TIME framework can be implemented to develop the high-tech remote patient monitoring dashboard in parallel with the high-touch dashboard alert protocol:

- *Plan:* we define the needs and scope for both the dashboard and the dashboard alert protocol by interviewing IMHP end users (mental health associates and nurses from each shift) from each inpatient unit to specify their needs and expectations.
- *Analyze:* we define the expected behavior and functionality of the monitoring dashboard and crisis intervention protocol based on what we learned from the IMHPs.
- *Prototype:* we create low-fidelity prototypes for both the dashboard and the dashboard alert protocol based on the requirements specified in the *Analyze* phase.
- *Test:* in a focus group, end users will interact with the monitoring dashboard and be trained on the dashboard alert protocol in a moderated usability study. The efficacy of the dashboard prototype and the implementability and instructional clarity of the dashboard alert protocol will be evaluated with defined key performance indicators (ie, time on task, system usability scale, conversion rate, and error rate).
- *Iterate:* we will use the IMHPs’ feedback from the *Test* phase to iterate high-fidelity prototypes for the monitoring dashboard and the dashboard alert protocol.
- *Implement:* we will again conduct focus groups to evaluate the monitoring dashboard and dashboard alert protocol in a simulated scenario within the target inpatient setting. The feedback will be used to inform further iteration or produce the final deliverables.

## Conclusions

The last few years have exposed an ongoing public mental health crisis through isolation, health anxiety, job loss, etc [45,46]. To meet these mental health needs—branded by increased public safety concerns and mental health issues—incorporating technology into mental health treatment is critical. At present, there is no established model for facilitating digital transformation—developing technology alongside a protocol for its eventual use—for provider-facing technology tools in inpatient psychiatry.

We propose an innovative model for designing a high-tech IMHP tool in parallel with a standardized, high-touch clinical protocol. Emphasizing the active involvement of the IMHP end user at each phase of the process will result in the most usable and acceptable digital intervention. The successful implementation of the TIME framework will provide an avenue for digital transformation in mental health care. To our knowledge, this is the first established model for facilitating digital transformation in inpatient psychiatric settings. Meeting the mental health needs of a postpandemic world by incorporating technology carefully into mental health ministration, especially for our most acute and intensive settings, will be lifesaving.

## Abbreviations

ACTS: Accelerated Creation-to-Sustainment
IMHP: inpatient mental health professional
SMI: severe mental illness
TIME: Technology Implementation for Mental-Health End-Users
